# BRD4 inhibition for the treatment of pathological organ fibrosis

**DOI:** 10.12688/f1000research.11339.1

**Published:** 2017-06-28

**Authors:** Matthew S. Stratton, Saptarsi M. Haldar, Timothy A. McKinsey

**Affiliations:** 1 Department of Medicine, Division of Cardiology and Consortium for Fibrosis Research & Translation, University of Colorado, Anschutz Medical Campus, Aurora, CO, USA; 2 Gladstone Institutes and Department of Medicine, Division of Cardiology, University of California San Francisco School of Medicine, San Francisco, CA, USA

**Keywords:** organ fibrosis, BRD4, anti-fibrotic therapies, small molecule BRD4 inhibitors, fibrotic diseases

## Abstract

Fibrosis is defined as excess deposition of extracellular matrix, resulting in tissue scarring and organ dysfunction. It is estimated that 45% of deaths in the developed world are due to fibrosis-induced organ failure. Despite the well-accepted role of fibrosis in the pathogenesis of numerous diseases, there are only two US Food and Drug Administration–approved anti-fibrotic therapies, both of which are currently restricted to the treatment of pulmonary fibrosis. Thus, organ fibrosis represents a massive unmet medical need. Here, we review recent findings suggesting that an epigenetic regulatory protein, BRD4, is a nodal effector of organ fibrosis, and we highlight the potential of small-molecule BRD4 inhibitors for the treatment of diverse fibrotic diseases.

## Introduction

**Figure 1.**
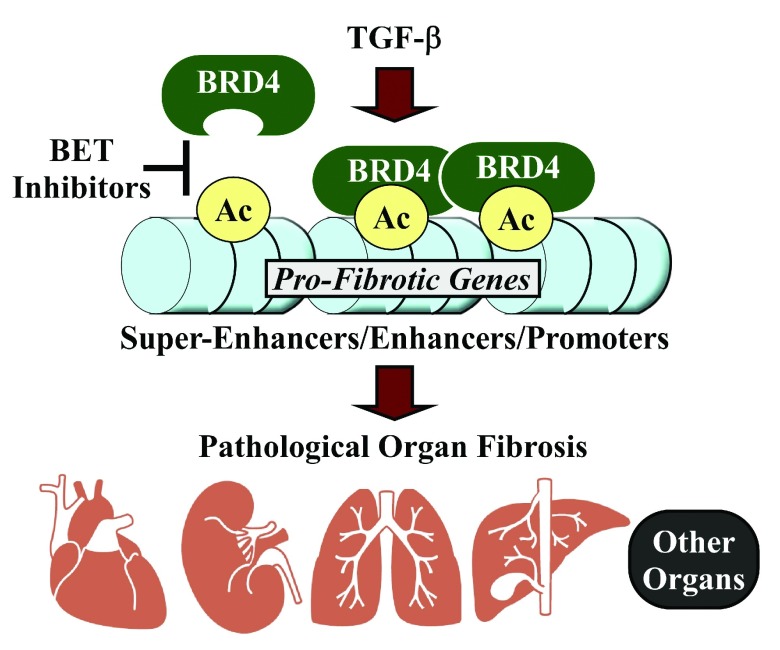
A model for the regulation of pathological organ fibrosis by BRD4. Stress signals, such as those elicited by transforming growth factor-beta (TGF-β), trigger recruitment of BRD4 to regulatory regions (super-enhancers, typical enhancers, and promoters) of genes that drive organ fibrosis. Bromodomain and extra-terminal (BET) inhibitors prevent association of BRD4 with these loci and thus suppress pro-fibrotic gene expression.

Acetylation of nucleosomal histone tails plays a fundamental role in epigenetic control of gene transcription. One mechanism by which acetylation regulates transcription is by creating docking sites for acetyl-lysine binding proteins, which often are referred to as “readers”. Among the most well-characterized proteins that bind acetyl-lysine marks on histone tails are the BET (bromodomain and extra-terminal) family of proteins, which consists of BRD2, BRD3, BRD4, and BRDT ^1, 2^. This review focuses on BRD4 because this BET family member has been clearly shown to regulate pro-fibrotic gene expression in various tissues. BRD4 associates with acetyl-histones via two tandem, amino-terminal bromodomains. The C-terminal region of BRD4 contains a carboxy-terminal motif (CTM) that is not present in other BET family members. Through its CTM, BRD4 interacts with protein complexes such as P-TEFb that signal to gene promoters. BRD4 directly associates with and allosterically activates cyclin-dependent kinase 9 (CDK9), the core kinase within the P-TEFb complex, resulting in phosphorylation of RNA polymerase II (Pol II) C-terminal heptapeptide repeats, and subsequent pause release and transcription elongation ( Figure 1) ^3^.

Knowledge of the functions of BET proteins in diverse physiological and pathophysiological processes has been greatly advanced by the availability of selective small-molecule BET inhibitors. These compounds—such as JQ1, I-BET151, and PFI-1—bind BET bromodomains with high affinity, thereby competitively displacing these epigenetic regulators from acetylated histones at target gene regulatory sites. BET proteins are promising therapeutic targets for a wide variety of diseases, and thus vigorous, ongoing medicinal chemistry efforts are aimed at developing newer generations of BET inhibitors with increased potency, selectivity, and drug-like properties ^4^. As detailed below, BET inhibitors have exhibited profound anti-fibrotic effects in rodent models of organ failure. Furthermore, genetic loss-of-function studies in cultured cells have revealed that BRD4 is a nodal, positive regulator of pro-fibrotic gene expression and that BRD4 promotes differentiation of precursor cells into myofibroblasts, a cell type that governs extracellular matrix (ECM) deposition and tissue remodeling throughout the body. We highlight these findings and expound on the promise of developing BET/BRD4 inhibitors for the treatment of fibrotic diseases that thus far have been recalcitrant to therapeutic intervention.

## BRD4 and pulmonary fibrosis

Pulmonary fibrosis is a devastating complication of a heterogeneous group of lung disorders collectively known as interstitial lung disease (ILD). Idiopathic pulmonary fibrosis (IPF), which is the most common ILD, manifests as progressive lung fibrosis that culminates in shortness of breath, dyspnea on exertion, and hypoxia. Strikingly, the median survival time following diagnosis of IPF is two to three years ^5^. The sole US Food and Drug Administration–approved anti-fibrotic therapies, which are pirfenidone (unknown mechanism of action) and nintedanib (tyrosine kinase inhibitor), are both indicated for the treatment of IPF. Unfortunately, the efficacy of these drugs is limited, and neither agent has been shown to significantly reduce mortality ^6, 7^.

In cultured lung fibroblasts (LFs) obtained from healthy donors, the BET inhibitors JQ1 and I-BET were shown to blunt induction of a multitude of pro-fibrotic, transforming growth factor-beta (TGF-β)-responsive genes, including alpha-smooth muscle actin (α-SMA), collagen 1A1, fibronectin, and interleukin-6 ^8^. Both compounds also blocked platelet-derived growth factor (PDGF)-mediated migration of LFs, a hallmark of the contractile myofibroblast state. Similar findings were made with LFs derived from patients with IPF ^9^. Importantly, BET inhibitor treatment of LFs did not directly inhibit TGF-β signaling, as SMAD activation was not directly affected. Rather, BET inhibition appeared to alter recruitment of BRD4 to histone H4K5 acetyl marks in regulatory elements of pro-fibrotic genes ^9^. More recently, using immortalized human small-airway epithelial cells, investigators found that BRD4 is required for TGF-β and nuclear factor-kappa B (NF-κB)/RelA-dependent expression of epithelial-mesenchymal-transition genes (for example, *SNAI1*, *TWIST1*, and *ZEB1*) by facilitating CDK9-mediated phosphorylation of RNA Pol II ^10^. In mouse models, JQ1 was found to suppress pulmonary fibrosis induced by bleomycin or repeated intranasal delivery of TGF-β, further validating a role for this epigenetic pathway in the control of lung fibrosis ^8– 11^.

## BRD4 and renal fibrosis

Chronic kidney disease (CKD) is caused by a heterogeneous group of conditions, such as diabetes and polycystic kidney disease. Remarkably, fibrosis is a final common pathway in the pathogenesis of all forms of kidney disease that lead to CKD. Clinical data have shown clearly that there is a correlation between the amount of renal fibrosis and progression of CKD ^12^. During kidney fibrosis, normal renal epithelial cells are replaced with inflammatory cells and fibroblasts, and there is increased deposition of ECM. As the normal architecture of the kidney erodes, renal failure progresses. In the US alone, more than nine million people have CKD and over 600,000 have end-stage renal disease, the most severe form of CKD (2015 USRDS Annual Data Report, Volume 2: End-stage Renal Disease; Centers for Disease Control and Prevention, Age-adjusted prevalence of CKD Stages 1–4 by Gender 1999–2012, CKD Surveillance Project).

Two recent articles suggest that BRD4 inhibition could be used as a strategy to treat renal fibrosis. In cultured normal rat or human kidney fibroblast cell lines (NRK-49F and HK-2), BET inhibitors blunted TGF-β-induced ECM gene expression, and this effect was recapitulated by BRD4 knockdown ^13, 14^. The BET inhibitor I-BET151 was shown to prevent renal fibrosis in a 7-day mouse unilateral ureteral obstruction (UUO) model ^13^, and JQ1 was able to attenuate progression of pre-existing renal fibrosis when delivered between days 7 and 14 post-UUO in rats ^14^. Interestingly, unlike what was observed in LFs, both BET inhibitors appeared to suppress renal TGF-β signaling, as indicated by reduced SMAD phosphorylation.

Inflammation is a key driver of fibrosis ^15^. In a comprehensive series of experiments, JQ1 was shown to potently inhibit renal inflammation in mice in response to UUO (up to 5 days) or angiotensin II infusion (3 days) or in a model of anti-glomerular basement membrane nephritis induced by nephrotoxic serum (NTS) administration (10 days) ^16^. The anti-inflammatory action of JQ1 was mediated, at least in part, by inhibition of NF-κB. In addition to suppressing inflammation, JQ1 prevented NTS-mediated renal functional impairment and improved glomerular lesions. It will be important to test BET inhibitors in more prolonged models of CKD where physiological markers of kidney function—such as serum creatinine, glomerular filtration rate, proteinuria, and water/electrolyte balance—are examined ^17^.

## BRD4 and hepatic fibrosis

Similar to CKD, fibrosis is the final common pathway of many chronic liver diseases, irrespective of etiology. Cirrhosis is the end-stage consequence of fibrosis of the hepatic parenchyma, resulting in altered hepatic biochemical function and impaired portal blood flow. Several clinically relevant complications arise from cirrhosis, including portal hypertension, encephalopathy, coagulopathy, peritoneal ascites, volume overload, hypoalbuminemia, susceptibility to infection, and increased risk of hepatocellular carcinoma. Cirrhosis affects hundreds of millions of patients worldwide, and liver transplantation remains the only long-term therapy for chronic cirrhosis that results in end-stage liver disease ^18^.

Hepatic stellate cells (HSCs) are the main cell type capable of pro-fibrotic transformation in the liver ^19^. In an RNA interference screen, BRD4 was found to be required for HSC activation in response to TGF-β ^20^. Consistent with this, three distinct BET inhibitors—JQ1, IBET-151, and PFI-1—blunted expression of nearly 30 fibrosis-related transcripts, and JQ1 suppressed HSC proliferation and differentiation into myofibroblasts, as evidenced by reduced α-SMA expression. Whole-genome chromatin immunoprecipitation sequencing (ChIP-seq) revealed that BRD4 associates with acetyl-histone H3K27-marked enhancers for a variety of pro-fibrotic genes in HSCs, and computational analyses suggested roles for pro-fibrotic transcription factors such as SRF, SMAD, and NF-κB in recruitment of BRD4 to these sites. Finally, JQ1 was also shown to be efficacious in a mouse model of liver fibrosis induced by carbon tetrachloride. Based on these findings, additional studies of BRD4 and BET inhibitors in the context of liver fibrosis are warranted ^21^.

## BRD4 and pancreatic fibrosis

Akin to the liver, the pancreas has resident stellate cells that produce high levels of ECM proteins upon activation ^22^. Pancreatic ductal adenocarcinoma (PDAC), a highly aggressive and deadly form of cancer, is characterized by dense, fibrotic ECM, which appears to enhance tumor progression ^23^. A recent report revealed a critical role for BRD4 in the control of pancreatic stellate cell (PSC) activation ^24^. In cultured PSCs, JQ1, I-BET151, and siRNA targeting BRD4 all reduced collagen mRNA and protein expression whereas knockdown of BRD2 and BRD3 appeared to enhance ECM expression. Crucially, in a mouse model of pancreatic cancer driven by transgenic expression of mutant kRas, JQ1 treatment led to blockade of myofibroblast differentiation and fibrosis within the pancreas. These findings suggest that BRD4 inhibition could be a useful strategy for the treatment of PDAC, as well as other forms of cancer such as myelofibrosis ^25^, where fibrosis contributes to disease progression ^26^.

## BRD4 and cardiac fibrosis

Increasing evidence implicates fibrosis as a key event that drives heart failure pathogenesis in response to stresses such as long-standing hypertension, myocardial infarction (MI), and aging ^27^. Heart failure affects nearly six million people in the US alone, 915,000 new cases are diagnosed annually, and the 5-year mortality rate is 42%, which exceeds that of many cancers ^28, 29^. In addition to contributing to contractile dysfunction, fibrosis disrupts normal patterns of cardiac electrical conduction, forming the substrate for arrhythmias and sudden cardiac death.

We previously showed that JQ1 prevents several hallmarks of heart failure, including cardiomyocyte hypertrophy, cardiac fibrosis, and systolic dysfunction, in a mouse model of aortic constriction-induced left ventricular pressure overload ^30– 32^. We found that, mechanistically, BRD4 promotes cardiomyocyte hypertrophy by triggering RNA Pol II pause release at promoters of pro-hypertrophic genes ^30^ and by contributing to the formation of long-range super-enhancers (SEs) associated with these genes ^33^; SEs are thought to signal to proximal promoters to stabilize coactivator complexes near transcription start sites and facilitate P-TEFb–mediated Pol II phosphorylation and transcription elongation ^34– 36^. Intriguingly, in addition to controlling pro-growth genes, many of the BRD4-enriched SEs identified in cardiomyocytes were associated with pro-fibrotic genes, including those encoding the secreted factors connective tissue growth factor (CTGF), plasminogen activator inhibitor-1 (PAI-1/Serpine1), and TGF-β2 ^33^. These findings suggest the possibility that BRD4 signaling in cardiomyocytes regulates expression of paracrine factors that crosstalk with fibroblasts and other stress-activated cell types in the heart to elicit fibrotic remodeling.

More recently, we have found that late administration of JQ1 also attenuates cardiac dysfunction both in the murine transverse aortic constriction (TAC) model and in post-MI cardiac remodeling ^37^. In addition, JQ1 blocked agonist-induced pathological hypertrophy and brain natriuretic peptide (BNP) expression in human induced pluripotent stem (iPS) cell-derived cardiomoycytes. Integrated transcriptomic analyses across rodent and human iPS cell models have made clear that JQ1 preferentially suppresses transactivation of a broad pro-fibrotic and pro-inflammatory gene program. Although precise delineation of cell type–specific effects *in vivo* will require the development of conditional and inducible genetic loss-of-function models for the BET alleles, the transcriptomic analysis of mouse bulk LV tissue revealed a strong suppression of myofibroblast activation by JQ1. Consistent with this, using cultured primary cardiac fibroblasts, we have found that BRD4 coordinates TGF-β–mediated pro-fibrotic gene expression and myofibroblast differentiation (unpublished data). Thus, BRD4 appears to play a crucial role in cardiac fibrosis by regulating fibrogenic gene expression in both cardiomyocytes and resident cardiac fibroblasts and possibly other cell types that populate the stressed myocardium.

## Conclusions

Organ fibrosis has devastating consequences, contributing to millions of deaths annually ^38^. The study of BRD4 and BET inhibitors in the context of fibrosis has just begun, and the mechanistic underpinnings of BRD4-dependent regulation of pro-fibrotic gene expression remain poorly understood. The encouraging pre-clinical findings highlighted above—particularly the ability of BET inhibitors to block pulmonary, renal, hepatic, pancreatic, and cardiac fibrosis in animal models—justify aggressive expansion of research and drug discovery efforts in this burgeoning arena.

There is reasonable concern about clinical evaluation of BET inhibitors without a more detailed understanding of the biological functions and mechanisms of action of distinct BET family members ^39^. We acknowledge that a significant amount of additional research is needed in this regard. However, given the extremely high mortality rate caused by organ fibrosis and the limited therapeutic options, we advocate for advancement of BET inhibitors into clinical trials for deadly fibrotic diseases, such as IPF. Furthermore, given the central role of BRD4 in the control of fibrosis across organ systems, BET inhibition is ideally suited to treat concomitant multi-organ fibrosis, such as occurs in cardiorenal syndrome, cardiopulmonary disease, and liver fibrosis resulting from the Fontan operation for single-ventricle congenital heart disease.

BRD4 was recently shown to regulate dermal myofibroblast differentiation, and BRD4 inhibition was found to suppress contracture of myofibroblasts isolated from humans with burn injury ^40^. Thus, it may be possible to establish proof-of-concept of anti-fibrotic action of BET inhibitors in humans by localized administration of compounds to skin to target hypertrophic scarring, prior to systemic delivery of the inhibitors for treatment of internal organ fibrosis. Nonetheless, more than 20 clinical trials with BET inhibitors for cancer applications are active or have been completed, and data from these trials should guide future evaluation of this compound class for the treatment of internal organ fibrosis. Furthermore, the feasibility of targeting global epigenetic regulators such as BET proteins for the treatment of human disease is bolstered by the fact that four histone deacetylase (HDAC) inhibitors are FDA-approved for the treatment of cancer ^41, 42^, that HDAC inhibitors have also shown promise in treating non-oncologic diseases such as Duchenne muscular dystrophy ^43^, and that HDAC inhibitors are fairly well tolerated in humans ^1^.

What is the mechanism? It is likely that BRD4 and BET inhibitors control organ fibrosis by regulating multiple gene programs and biochemical pathways in diverse cell types. We favor a model in which BET inhibitors block dynamic BRD4 association with subsets of specific enhancers and promoters that regulate transcription of downstream genes encoding ECM proteins and factors that stimulate myofibroblast differentiation ( Figure 1). Furthermore, it is probable that BET inhibitors indirectly suppress pro-fibrotic signaling networks, as was observed for ERK and SMAD in the kidney ^14, 15^. It is also highly likely that BET inhibition extends beyond fibroblasts/myofibroblasts to control fibrosis. For example, BET inhibitors have potent anti-inflammatory activity ^2, 44^, and inflammation is thought to be a key driver of fibrosis ^16^. Regardless of the precise mechanism(s) by which these epigenetic factors regulate fibrosis, the compelling in vivo validation data obtained with BET inhibitors in models of organ fibrosis leads us to conclude that the field should double down on BETs for the treatment of fibrotic diseases in humans. 
